# Physiologically Based Biopharmaceutics Modeling of Regional and Colon Absorption in Dogs

**DOI:** 10.1021/acs.molpharmaceut.0c01201

**Published:** 2021-03-15

**Authors:** Emma Eckernäs, Christer Tannergren

**Affiliations:** Oral Product Development, Pharmaceutical Technology & Development, Operations, AstraZeneca, Gothenburg, S-431 83 Mölndal, Sweden

**Keywords:** physiologically based biopharmaceutics modeling, PBPK, PBBM, drug absorption, colon absorption, in silico prediction

## Abstract

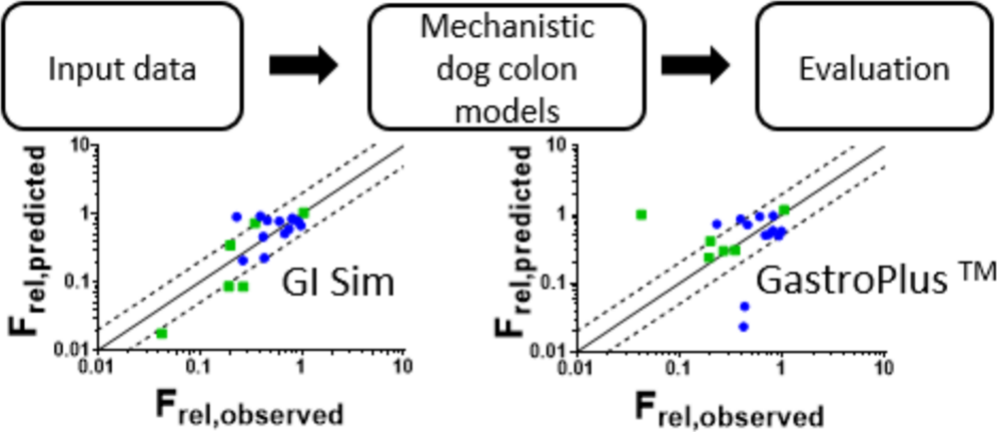

Colon absorption is a key determinant for the successful development of modified-release (MR) formulations, and the risk that colon absorption may limit the in vivo performance of an MR product can be assessed early by various in vitro tests or by preclinical in vivo regional absorption studies in dogs. Mechanistic physiologically based biopharmaceutics modeling (PBBM) is becoming increasingly accepted to predict in vivo performance and guide formulation development; however, no evaluation of the ability to predict colon absorption has been performed. The purpose of this study was to investigate if regional and colon absorption of drugs in dogs could be predicted with sufficient accuracy using PBBM to enable the replacement of in vivo dog studies in the early assessment of colon absorption limitation risks. This was done by predicting the regional and colon absorption and plasma exposure of 14 drugs after administration to the dog colon according to an a priori approach using the in silico absorption models GI-Sim and GastroPlus. Predictive performance was primarily assessed by comparing observed and predicted plasma concentration–time profiles, AUC_0-*t*_, and the relative bioavailability in the colon (*F*_rel,colon_) as compared to an oral/duodenal reference. Trends in dependency of prediction performance on predicted fraction absorbed, permeability, and solubility/dissolution rate were also investigated. For GI-Sim, the absolute average fold error (AAFE) values for AUC_0-*t*_ and *F*_rel,colon_ were within a 2-fold prediction error for both solutions (1.88 and 1.51, respectively) and suspensions (1.58 and 1.99, respectively). For GastroPlus, the AAFE values for AUC_0-*t*_ and *F*_rel,colon_ were outside the set 2-fold prediction error limit for accurate predictions for both solutions (3.63 and 2.98, respectively) and suspensions (2.94 and 2.09, respectively). No trends for over- or underprediction were observed for GI-Sim, whereas GastroPlus showed a slight trend for underprediction of both AUC_0-*t*_ and *F*_rel,colon_ for compounds with low permeability. In addition, regional differences in the plasma profiles were qualitatively predicted in the majority of cases for both software. Despite the differences in prediction performance, both models can be considered to predict regional differences in absorption as well as AUC_0-*t*_ and *F*_rel,colon_ with acceptable accuracy in an early development setting. The results of this study indicate that it is acceptable to replace in vivo regional absorption studies in dogs with the evaluated models as a method for the early assessment of the risk for colon absorption limitation of MR drug product candidates.

## 1 Introduction

Gastrointestinal (GI) absorption is one of the key factors determining the in vivo performance of orally administered drugs. GI permeability and solubility of the drug as well as the dissolution/release rate from the formulation are the main determinants of the fraction of the dose absorbed.^1^ The small intestine (SI) is usually viewed as the main site for drug absorption, but for modified-release (MR) formulations and for drugs with insufficient SI absorption, absorption from the colon must be considered. Consequently, it is important to understand the colon absorption for such drug candidates.^2^ As the colon is structurally and anatomically different from the SI, it provides additional barriers against drug absorption. Differences in permeability between the SI and the colon due to smaller surface area and tighter junctions in the epithelial cell layer have been reported and differences in transporter expression levels may also result in regional permeability differences.^3−5^ Furthermore, factors including lower water content, irregular motility, viscosity, and lack of bile salts are believed to restrict solubility and dissolution in the colon.^6,7^ The distribution of drug-metabolizing enzymes has also been reported to vary between regions and drugs may be subject to bacteria-mediated degradation in the colon.^8−10^ It is of great importance to understand the impact of regional differences in intestinal absorption as well as to be able to predict the extent of absorption from the colon and consequently the in vivo performance of MR products.^2^

The extent of colon absorption in humans may be assessed directly by human regional relative bioavailability studies using intubation, capsule techniques, and colonoscopy techniques.^4,11−14^ Usually these studies are performed before initiating MR product development, but ideally the development risks associated with limited colon absorption should be assessed early during the candidate selection or preclinical development phases. Recently, in vivo predictive in vitro methods such as in vitro permeability assays, simulated biorelevant colon media for solubility/dissolution investigations as well as colon stability assays have emerged as tools for the early assessment of the potential for absorption in the colon.^2,3,9,10,15−17^ In addition, it has been demonstrated that dog colonoscopy and colon stoma models can be predictive of human colon absorption and permeability, and as a result, the dog is currently the main preclinical model for the assessment of colon absorption limitation risks.^5,18−21^

Despite recent advancements, the colon absorption assessment capability could be further improved. In vivo studies are costly and time consuming, and in addition, there are ethical aspects to consider for animal in vivo studies, where the aim should be to remove or replace such studies with other methodologies when possible. In addition, the available in vitro methods all have the limitation that they only measure one parameter in isolation. Recently, the application of mechanistic physiologically based biopharmaceutics modeling (PBBM)^22^ has become increasingly acceptable for predictions of the rate and extent of absorption. There are several software packages available for the prediction of intestinal absorption such as GastroPlus, Simcyp, PK-Sim, and GI-Sim.^23−26^ These models integrate anatomical and physiological parameters, physicochemical properties of the active pharmaceutical ingredient as well as formulation properties to predict the in vivo performance of a drug.^27^ The models have the advantage of being able to incorporate all aspects of importance for absorption thus enabling a potential comprehensive assessment of a drug candidate. There are several cases where absorption modeling has also been proven useful to guide MR formulation development.^18,28,29^ Furthermore, in silico models of preclinical species have been used to improve the confidence in predictions of human regional absorption.^30^ To successfully apply these models in drug development in the absence of any measured in vivo data, the ability of the in silico models to adequately predict in vivo performance should first be evaluated. Recently, evaluations of the predictive performance of several available models with respect to absorption mainly in the SI have been published.^26,31,32^ However, the need for improved colon models has been identified and an in-depth evaluation of the predictive power regarding colon absorption has not been published.^2,31,33^

The main purpose of this study was to investigate the ability of GI-Sim and GastroPlus to predict the regional and colon absorption of drugs in dogs to evaluate if PBBM approaches could be used to replace dog in vivo studies in the early assessment of colon absorption limitation risks. This would in turn reduce the use of animals and enable a more time and cost-efficient MR product development.

## 2 Methods and Materials

### 2.1 Modeling Strategy

The predictive performance of the dog colon models in GI-Sim and GastroPlus were evaluated through predictions of fraction absorbed (*f*_abs_), the relative colon bioavailability (*F*_rel,colon_), and plasma pharmacokinetic (PK) parameters, primarily area under the plasma concentration–time curve (AUC), for a set of model drugs, which have been administered both orally (or to the duodenum) and directly to the colon in dogs. The study included simulations of 14 compounds, administered as solutions and/or suspensions. The absorption modeling was performed according to an a priori approach where no fitting to observations was allowed, while the systemic PK input parameters were obtained by compartmental modeling of intravenous data. An effort was taken to harmonize the input parameters between the different software. In vivo data from different dog breeds were used, including data from Beagle, Labrador, and Mongrel dogs.

### 2.2 Investigated Absorption Models

The two different software evaluated in this study were GastroPlus (version 9.0.0007) and GI-Sim (version 5.2). They both employ a series of coupled compartments as a model of the GI tract.^23,26^ The compartments are defined by parameters such as surface area, luminal pH, and fluid volume to mimic the physiological environment. For this evaluation, the fasted Beagle physiology model in GastroPlus was used, while the fasted Beagle physiology model in GI-Sim was refined to allow colon absorption modeling (see Section 2.2.1).

#### 2.2.1 GI-Sim

GI-Sim is a mechanistic physiologically based absorption model, which has been internally developed at AstraZeneca and has been thoroughly described elsewhere.^26^ The fasted Beagle physiology in GI-Sim consists of nine compartments: stomach (1), duodenum (2), jejunum 1 (3), jejunum 2 (4), ileum 1 (5), ileum 2 (6), ileum 3 (7), ileum 4 (8), and colon (9). For the purpose of this study, the surface area in the colon compartment in the dog model was derived from the GI-Sim human fasted model. In the human model, the colonic surface area (including the cecum) constitutes 3.5% of the total surface area in the GI tract. Assuming that the same is true for the dog, a colon surface area of 17 cm^2^ was estimated. This area was not intended to reflect the true physiological area of the dog colon but rather an initial estimate of the area available for absorption. The full physiological model, including the updated surface area, is described in Table 1. Simulation of absorption after colon administration was achieved using a dose-to-colon module, where the drug is administered directly to the colon compartment. Thus, it was not necessary to adjust the transit times and fluid volumes in the stomach and the SI compartments. Simulation of reference administrations to the duodenum was simulated by administration directly to the duodenum compartment, whereas oral administrations were simulated without any adjustments to the model. The “solution” and “suspension” formulation options were selected for solutions and suspensions, respectively. In accordance to the previously described standard procedure, absorption in the colon was not allowed for predictions of oral/duodenal (reference) administrations.^26,31^ Since the dose-to-colon option currently does not allow entry of particle size distribution data, only mean particle radius was used as input in the GI-Sim predictions.

**Table 1:** Summary of the Updated Fasted Beagle Physiology in GI-Sim

GI-compartment	surface area (cm^2^)	volume (mL)	transit time (min)	pH	micellar volume fraction
stomach	0	450	15	3.0	0
duodenum	140.6	35.16	15.6	6.2	0.0002
jejunum 1	103.6	25.90	15.6	6.2	0.0002
jejunum 2	76.3	19.08	15.6	6.2	0.0002
ileum 1	56.2	14.06	15.6	6.4	0.0002
ileum 2	41.4	10.36	15.6	6.6	0.0002
ileum 3	30.5	7.632	15.6	6.68	0.0002
ileum 4	22.5	5.621	15.6	6.75	0.0002
colon	17	78.50	720	6.45	0

#### 2.2.2 GastroPlus

GastroPlus (Simulations Plus, Inc., Lancaster, CA) is based on the advanced compartmental absorption and transit (ACAT) model and has previously been described by Agoram et al.^23^ The “immediate release solution” or “immediate release suspension” dosing options were used for solutions and suspensions, respectively. For AZ1, particle size was described by fitting a distribution curve onto the full particle size distribution using 10 particle size bins. For all other compounds, mean particle size was used as input. The fasted Beagle physiology in GastroPlus is made up of nine compartments: stomach (1), duodenum (2), jejunum 1 (3), jejunum 2 (4), ileum 1 (5), ileum 2 (6), ileum 3 (7), cecum (8), and ascending colon (9). The physiology is summarized in Table 2. To simulate administration directly to the colon, the transit times in compartments 1–7 were set to 0.001 min and the % fluid in SI was set to 0.1. Oral and duodenal reference administrations were simulated using default settings or by setting the transit time in compartment 1 to 0.001 min, respectively.

**Table 2:** Summary of the Default Fasted Beagle Physiology in GastroPlus

GI-compartment	length (cm)	radius (cm)	SEF^a^	volume (mL)	transit time (min)	pH	bile salt (mM)
stomach	15.00	1.00	1.000	51.00	15	3.00	0.0
duodenum	12.43	0.62	6.940	6.083	16.8	6.20	5.000
jejunum 1	66.64	0.47	5.905	18.58	51	6.20	4.050
jejunum 2	66.64	0.41	4.161	13.74	37.8	6.20	1.820
ileum 1	1.43	0.47	3.271	0.389	1.2	6.40	0.610
ileum 2	1.43	0.47	3.233	0.396	1.2	6.60	0.440
ileum 3	1.43	0.47	3.196	0.403	1.2	6.68	0.310
cecum	1.99	0.93	1.630	0.538	228.6	6.75	0.0
Asc colon	4.26	1.42	1.700	2.700	491.4	6.45	0.0

a Surface area enhancement factor.

### 2.3 Model Drug Selection and Data Collection

The selection of model drugs in this investigation was based on the availability of in vivo data after administration directly to the colon in dogs. An effort was made to include a broad range of compounds, covering all four BCS classes. Systemic PK parameters were estimated by compartmental modeling of the plasma profiles after intravenous administration using the PK Plus module in GastroPlus (Table 3). All plasma concentration data were gathered either from previously published work or, where no reference is indicated, from studies performed in house at AstraZeneca. A general description of the methodology used to investigate the regional absorption of AZ1, AZ2, and AZ3 in dogs has been described earlier.^5^ When intravenous and oral/colon data for a specific compound were obtained from different dog breeds, the PK parameters (i.e., clearance and volumes of distribution) were normalized against body weight to reflect the correct breed in the predictions of exposure after oral/colon administrations. The same PK parameters were used as input in GI-Sim to avoid potential differences in PK algorithms. The first-pass liver extraction was estimated by
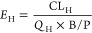
where *E*_H_ is the hepatic extraction ratio, CL_H_ the hepatic clearance, *Q*_H_ the hepatic blood flow (39.6 L/h for a 12 kg dog), and B/P is the blood:plasma concentration ratio (B/P = 1 in all simulations). CL_H_ was assumed to be equal to nonrenal clearance and was calculated by CL = CL_H_ + CL_R_. CL_R_ was estimated by fu × GFR, where fu is the fraction unbound and GFR is the glomerular filtration rate, which was assumed to be 61.3 mL/min for a 12 kg dog. Where no values for fu were available (ketoprofen and enalaprilat), CL_R_ was assumed to be zero. For metoprolol, the estimated CL_R_ using the abovementioned strategy generated first-pass values above 100%. For this reason, *Q*_H_ was normalized against the weight of 30 kg dog, and this value (99 L/h) was used to estimate the first-pass extraction of metoprolol. For theophylline, the calculated CL_R_ was higher than the total CL generated by compartmental modeling of the available in vivo data, and in this case, CL_R_ was set at zero for the purpose of the simulations. Biopharmaceutics and physicochemical properties of the drugs were gathered from previously published reports or internal measurements at AstraZeneca (Table 4). In vitro solubility in buffer and fasted simulated small intestinal fluid (FaSSIF) were used when available. Solubility was assumed to be the same in FaSSIF as in buffer when no biorelevant solubility was available, i.e., no partitioning into micelles was assumed. Missing particle size data was handled by assuming a mean particle diameter of 20 μm as previously described.^26,31^ Molar density (ρ) was calculated by ρ *=M*_W_/*V*_M,_ where *M*_W_ is the molecular weight and *V*_M_ is the molar volume. The diffusion coefficient in water (*D*) was estimated by Stoke–Einstein’s equation
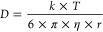
where *k* is the Boltzmann’s constant, *T* is the absolute temperature, η is the viscosity of water, and *r* is the molecule radius. Missing data on ρ or *D* were handled using default values of 1.2 g/mL and 0.76 × 10^–9^ m^2^/s, respectively, as previously described.^26,31^ The effective permeability (*P*_eff_) in dogs was estimated as described below.

**Table 3:** Systemic Compartmental Pharmacokinetic Parameters and Fraction Lost during First-Pass Used in the Simulations

	**CL** (L/h/kg)	**V** (L/kg)	***k*_12_** (h^–1^)	***k*_21_** (h^–1^)	***k*_13_** (h^–1^)	***k*_31_** (h^–1^)	**fu**^a^	**first-pass extraction** (%)
Aprepitant	0.09	0.204	6.887	2.722	n/a	n/a	0.014	2.74
Atenolol	0.268	0.97	1.29	0.584	n/a	n/a	0.9	3.45
AZ1	0.467	0.116	27.82	6.96	4.839	1.039	0.063	35.35
AZ2	0.086	0.366	0.073	0.011	n/a	n/a	n/a	2.59
AZ3	0.624	0.364	7.153	2.369	n/a	n/a	0.0022	47.25
Cimetidine^35^	0.714	0.424	5.909	3.472	0.218	0.323	0.9	21.12
Enalaprilat	0.155	0.751	1.149	0.338	n/a	n/a	n/a	0.47
Felodipine^36^	1.142	0.65	n/a	n/a	n/a	n/a	0.001	40.53
Ketoprofen	0.146	0.158	2.018	1.76	0.394	0.188	n/a	11.03
Metoprolol	2.643	8.92	n/a	n/a	n/a	n/a	0.85	53.0
Nifedipine^37^	2.638	1.368	2.709	0.69	n/a	n/a	0.076	79.95
Propranolol^38^	0.934	1.087	5.828	2.782	1.887	0.09	0.19	62.0
Ranitidine^39^	0.60	0.13	7.942	1.215	0.289	0.141	0.71	13.69
Theophylline^40^	0.083	0.558	n/a	n/a	n/a	n/a	0.85	2.51

a Fraction unbound used in simulations.

**Table 4:** Biopharmaceutics and Physicochemical Input Parameters of the Model Compounds Included in the Evaluation

	*M*_w_ (g/mol)	p*K*_*a*_^a^	log* D*_7.4_	ρ (g/mL)	particle radius^b^ (μm)	*D* (10^–9^·m^2^/s)	*P*_eff,dog_^c^ (10^–4^·cm/s)	*S*_buffer_ (pH) (μg/mL)	*S*_FaSSIF_ (μg/mL)	BCS
Aprepitant	534	9.15 a	6.9^31^	1.51^31^	0.12	0.63^31^	7.1^31^	0.37 (6.5)^31^	23^31^	II
		2.4 b^31^								
Atenolol	266	9.21 b^26^	–2^5^	1.1		0.72^26^	0.82^5^	13 300 (intrinsic)^2^	13 300^d^	III
AZ1	450 ± 5	12 a	2.9	1.38	25	0.68	4.16	8.9 (6.5)	17	II
		2.2 b								
AZ2	400 ± 5	11 a	1.36	1.38	5	0.68	3.92	253 (6.5)	253	IV
AZ3	520 ± 5	3.05 b	3.89	1.24	5	0.60	6.9	7 (7.4)	360	IV
Cimetidine	252	6.76 b^26^	0.23	1.15		0.77^26^	1.03^26^	24 000 (6.8)^2^	24 000^d^	III
Enalaprilat	348	7.84 a	–1^5^			0.69^26^	0.82^5^	5000 (water)^41^	5000^d^	III
		3.17 b^5^								
Felodipine	384	neutral^31^	4.3^31^	1.28^31^		0.67^31^	7.7^31^	1 (6.5)^31^	53^31^	II
Ketoprofen	254	4.02 a^26^	0.1^5^	1.14		0.75^26^	8.7^26^	51 (1.2)^42^	51^d^	II
Metoprolol	267	9.18 b^26^	0^5^	1.07		0.71^26^	4.83^26^	43 000 (6.5)^2^	43 000^d^	I
Nifedipine	346	neutral^20^	2.07^20^				3.6^43^	11 (6.5)^20^	17^43^	II
Propranolol	259	9.4 b^20^	1.16			0.72^26^	2.91^26^	1000 (6.5)^20^	1000^d^	I
Ranitidine	351	7.62 b	–0.94	1.15		0.69^26^	0.80^44^	1750 (7.4)	1750^d^	III
		2.22 b								
Theophylline	180	8.4 a	–0.14	1.25		0.85	7.2	1800 (7.4)	1800^d^	I

a For *p*K_a_ values, the notations a and b represent acid and base, respectively.

b Particle size is presented as a mean particle radius. For AZ1, the full particle size distribution was used as input in the models.

c Estimated dog *P*_eff_ applied in the simulations.

d The same value as *S*_buffer_ due to the lack of FaSSIF solubility data.

#### 2.3.1 Strategy to Estimate the Effective Permeability in Dogs

Since dog intestinal *P*_eff_ values are rarely available and there is no well-established correlation between dog and human *P*_eff_, three different strategies were evaluated to estimate *P*_eff_ in dog in this study:

1. The first approach assumed that *P*_eff_ is the same in dogs and humans for all compounds.
2. The second approach used the correlation incorporated in GI-Sim, which assumes that *P*_eff_ in dogs is approximately 3 times higher than the human *P*_eff_. In GastroPlus, the dog *P*_eff_ is approximately 2.4–3-fold higher than the human *P*_eff_, depending on the permeability input value. Therefore, approach 2 was considered to be representable for the default settings in GastroPlus.
3. The third strategy divided the compounds into two groups based on previous work by Dahlgren et al.^5^ Their results indicate that *P*_eff_ is higher in dog for low-permeability compounds, but that *P*_eff_ is similar in dogs and humans for high-permeability compounds.^5^ In this evaluation, a limit was set at a human *P*_eff_ of 1.34 (human *P*_eff_ of the high-permeability marker metoprolol). Compounds with a *P*_eff_ lower or equal to 1.34 were assumed to follow the GI-Sim correlation of having a 3-fold higher permeability in dogs. Compounds with a *P*_eff_ above 1.34 were assumed to have the same *P*_eff_ in dogs and humans.

Previously measured or estimated human *P*_eff_ values were used as a basis for all three approaches. Where no *P*_eff_ values were available, apparent permeability (*P*_app_) in Caco-2 cell lines were used to predict human *P*_eff_ according to a previously established Caco-2 *P*_app_–human *P*_eff_ correlation. The different approaches were evaluated in initial simulations of 15 compounds after oral and colon administrations in GI-Sim only. A strategy was chosen based on the ability to predict area under the plasma concentration–time curve up to the last measured concentration (AUC_0-*t*_), peak plasma concentration (*C*_max_), and time to peak plasma concentration (*t*_max_) and was used for the full evaluation in both software.

### 2.4 Prediction Performance Assessment

The evaluation of the ability of the models to predict the extent of absorption in the colon was primarily based on the ability to predict the mean AUC_0-*t*_ and the relative bioavailability after administration to the colon (*F*_rel,colon_) in comparison to oral/duodenal administration (AUC_colon_/AUC_ref_). The predicted fraction absorbed in the colon (*f*_abs,colon_) was also noted for each simulation. The absolute average fold error (AAFE) was used as a measure of the overall predictive accuracy.
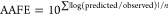
Using the ratio of absolute predicted and observed values, over- and underpredictions will not cancel each other out and AAFE will consequently serve as a measure of the overall accuracy. To assess the tendency for over- or underprediction, the average fold error (AFE) was used.
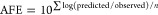
AFE values below 1 indicate a trend for underprediction, whereas values above 1 indicate overprediction. A model with perfect accuracy and no systematic trend for over- or underprediction would hence have both AAFE and AFE values of 1. A AAFE ≤ 2, i.e., a 2-fold prediction error, was defined as accurate in this evaluation, which is in accordance with the prediction criteria for other PK parameters at the stage of development as considered here.^34^ Furthermore, the percentage of the predictions within 2-fold of the observations were documented.

Results were examined to discover any trends in the predictive performance depending on *P*_eff_, solubility, or predicted *f*_abs, colon_.

## 3 Results

### 3.1 Selection of Strategy to Estimate *P*_eff_ in Dogs

Out of the three evaluated strategies to estimate *P*_eff_ in dogs, the strategy which divided the compounds into two different groups according to permeability class was found to be somewhat better than the other approaches and was selected for estimation of dog *P*_eff_ throughout the remainder of the study (AAFE_AUC_ = 1.84, AFE_AUC_ = 1.08). The strategy assuming dog *P*_eff_ = human *P*_eff_ resulted in a tendency for underprediction of AUC (AAFE_AUC_ = 2.30, AFE_AUC_ = 0.78), whereas the strategy assuming 3-fold higher *P*_eff_ in dogs compared to humans regardless of permeability class resulted in a tendency for overprediction (AAFE_AUC_ = 2.10, AFE_AUC_ = 1.54). The estimated dog *P*_eff_ values used in the final simulations are summarized in Table 4.

### 3.2 Evaluation of Colon Absorption Prediction Performance

**Figure 1:**
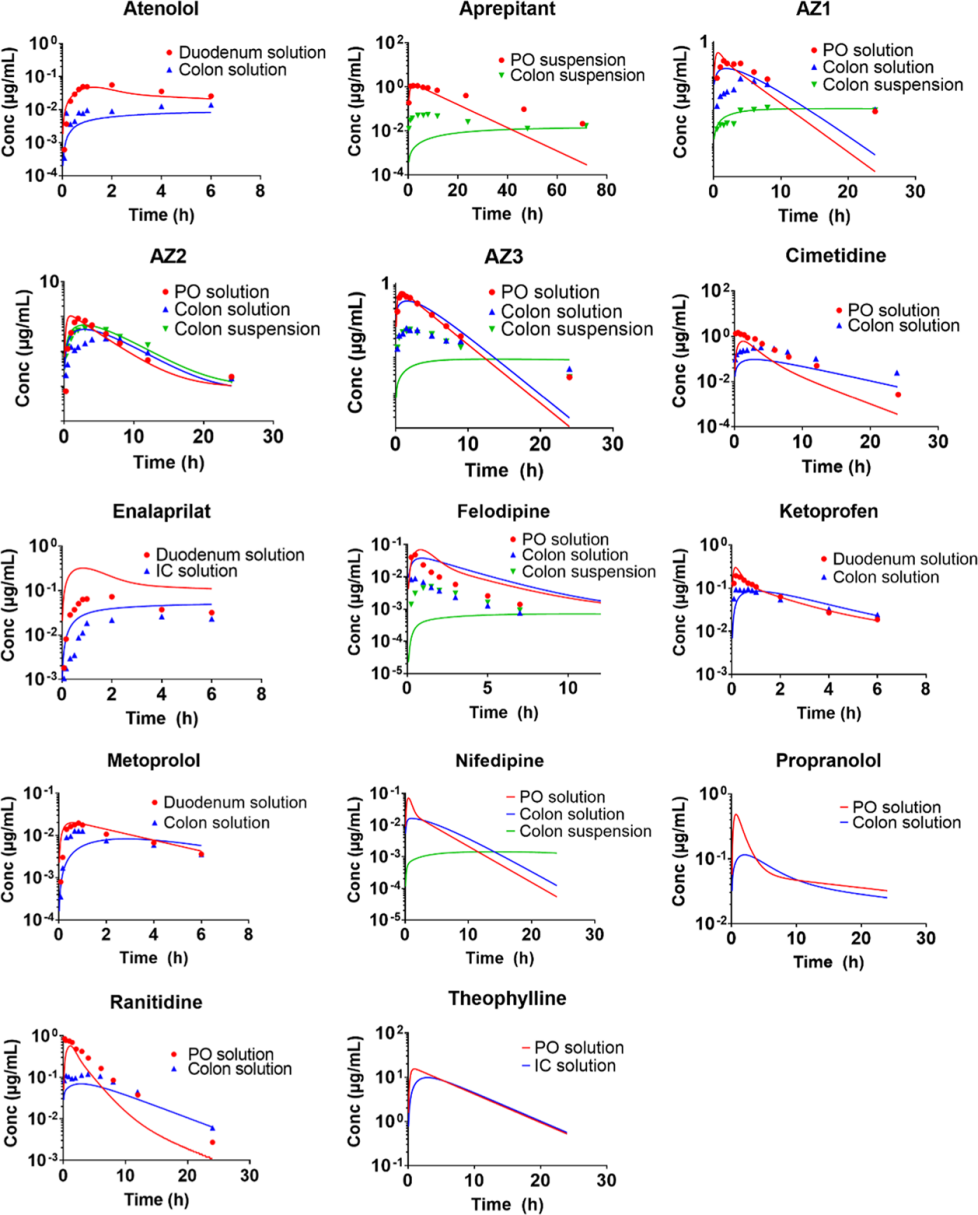
Mean observed and predicted plasma concentration–time profiles after oral/duodenal and colon administration using GI-Sim. Observed data is depicted with symbols and predicted data with solid lines. No observed plasma concentration–time profiles were available for theophylline, nifedipine, and propranolol.

**Figure 2:**
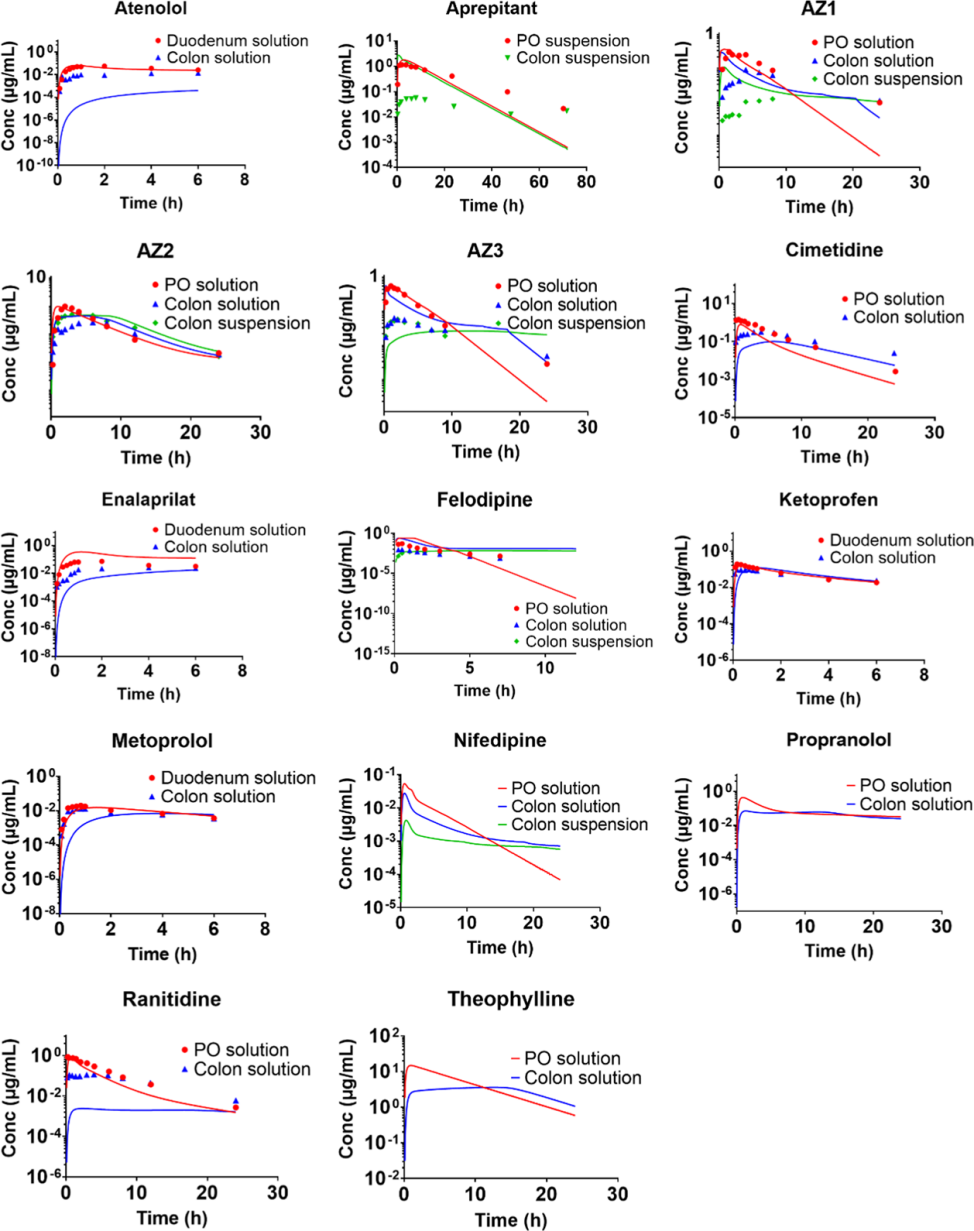
Mean observed and predicted plasma concentration–time profiles after oral/duodenal and colon administration using GastroPlus. Observed data is depicted with symbols and predicted data with solid lines. No observed plasma concentration–time profiles are available for theophylline, nifedipine, and propranolol.

**Figure 3:**
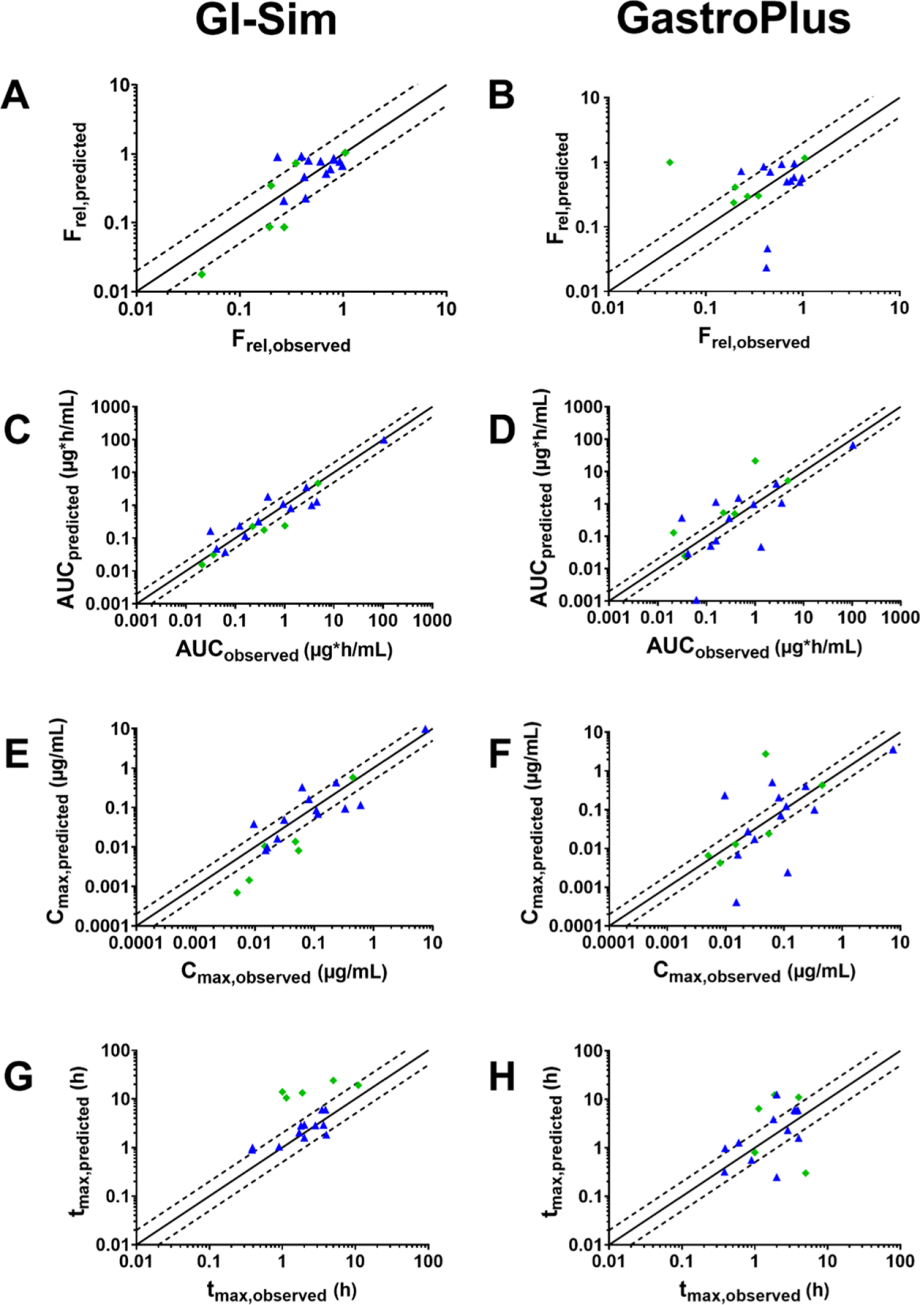
Colon absorption prediction performance of *F*_rel,colon_, AUC_0-*t*_, *C*_max_, and *t*_max_ for solutions (blue triangles) and suspensions (green diamonds) after direct administration to the colon in dogs. GI-Sim results are displayed in the left column and GastroPlus in the right column. The solid line is the line of unity and the dotted lines represent a 2-fold deviation.

GI-Sim and GastroPlus were primarily evaluated with respect to their ability to predict AUC_0-*t*_, and *F*_rel,colon_ after administration to the colon in dog, but also with regards to *C*_max_ and *t*_max_. Thirteen of the 14 model drugs were administered to the colon as a solution, while colon absorption data for suspensions were available for six of the model drugs. The observed and predicted plasma concentration–time profiles after oral/duodenal and colon administration are shown in Figures 1 and 2 for GI-Sim and GastroPlus, respectively. A summary of observed and predicted data is presented in Table 5. The overall predictive performance of both software is summarized in Table 6 and Figure 3.

**Table 5:** Observed and Predicted Dog Colon Absorption Parameters of the Model Drugs in Relation to Dose and Formulation Type Applied in the Simulations

			AUC_0-*t*_^a^ (μg × h/mL)	AUC_0-*t*,pred_^b^ (μg × h/mL)		*F*_rel, colon_^c^	*F*_rel,colon,pred_^d^		*f*_abs,colon,pred_^e^	
drug	dose (mg)	formulation	obs	GIS	G+	obs	GIS	G+	GIS	G+
Aprepitant^45^	24	nanosuspension	1.01	0.24	21.5	0.04	0.02	1.00	1.6	100.0
Atenolol^5^	5	solution	0.06	0.04	0.00	0.27	0.21	0.01	23.1	3.0
AZ1	30	solution	0.94	1.10	0.98	0.46	0.80	0.71	79.6	71.7
AZ1	40	suspension	0.22	0.23	0.53	0.20	0.35	0.41	13.6	30.2
AZ2	15	solution	2.76	3.52	4.21	0.61	0.78	0.94	78.6	94.6
AZ2	20	suspension	4.76	4.68	5.23	1.05	1.05	1.17	78.4	88.4
AZ3	75	solution	0.46	1.83	1.53	0.23	0.91	0.73	86.6	72.9
AZ3	75	suspension	0.38	0.18	0.50	0.19	0.09	0.24	9.1	25.9
Cimetidine	87	solution	3.55	1.00	1.09	0.68	0.52	0.50	46.2	50.2
Enalaprilat^5^	20	solution	0.12	0.24	0.05	0.43	0.23	0.05	25.6	10.2
Felodipine	10	solution	0.03	0.17	0.37	0.39	0.93	0.86	87.8	86.1
Felodipine	10	suspension	0.02	0.02	0.13	0.27	0.09	0.30	9.1	30.1
Ketoprofen^5^	2.5	solution	0.29	0.32	0.36	0.82	0.83	0.96	89.6	99.2
Metoprolol^5^	12.5	solution	0.04	0.05	0.03	0.75	0.60	0.52	73.7	70.4
Nifedipine^20^	24	solution	0.16	0.12	0.07	0.93	0.78	0.49	77.1	50.3
Nifedipine^20^	12	suspension	0.04	0.03	0.02	0.35	0.73	0.30	22.7	17.2
Propranolol^20^	48	solution	4.51	1.27	1.15	0.98	0.67	0.58	70.7	64.1
Ranitidine	63	solution	1.33	0.81	0.05	0.42	0.46	0.02	38.9	2.7
Theophylline^20^	120	solution	104	98.8	66.7	0.81	0.86	0.59	87.1	62.8

a Area under the curve between time zero and the last observed time point.

b Predicted area under the curve between time zero and the last observed time point.

c Relative bioavailability after administration to colon as compared to oral/duodenal administration.

d Predicted relative bioavailability after administration to colon as compared to oral/duodenal administration.

e Predicted fraction absorbed in colon.

**Table 6:** Summary of the Predictive Performance of GI-Sim and GastroPlus After Colon Administration in Dogs^a^

			% predictions (*n*) within 2-fold deviation	AAFE	AFE
	solutions	GI-Sim	69 (9)	1.88	1.04
AUC		GastroPlus	38 (5)	3.63	0.54
	suspensions	GI-Sim	67 (4)	1.58	0.64
		GastroPlus	50 (3)	2.94	2.59
	solutions	GI-Sim	85 (11)	1.51	1.10
*F*_rel_		GastroPlus	54 (7)	2.98	0.53
	suspensions	GI-Sim	33 (2)	1.99	0.77
		GastroPlus	67 (4)	2.09	1.99

a Results are shown as a percentage of simulations that fall within each specific accuracy level, as well as the absolute average fold error (AAFE) and average fold error (AFE).

For solutions in GI-Sim, the AAFE values for AUC_0-*t*_ and *F*_rel,colon_ were both within a 2-fold prediction error (1.88 and 1.51, respectively) and there was no trend for over-/underprediction with corresponding AFE values of 1.04 and 1.10, respectively (Table 6). The predictions of AUC_0-*t*_ and *F*_rel,colon_ were within a 2-fold deviation from the observed values in 69 and 85% of the cases, respectively, for the solutions (Table 6). Similarly, for suspensions, the AAFE values for both AUC_0-*t*_ and *F*_rel,colon_ were within a 2-fold prediction error (1.58 and 1.99, respectively), but the corresponding AFE values of 0.64 and 0.77 indicated a trend for underprediction (Table 6). The predictions of AUC_0-*t*_ for the suspensions were within a 2-fold deviation from the observed values in 67% of the cases, while *F*_rel,colon_ predictions were only within that range for 33% of the cases (Table 6). Predictions of *C*_max_ and *t*_max_ were within a 2-fold deviation from the observed values in more than 50% of the cases for solutions (Figure 3). For suspensions, *C*_max_ tended to be underpredicted whereas *t*_max_ was generally overpredicted (Figure 3). Overall, the simulated and observed plasma profiles (Figure 1) agreed well and regional differences in absorption were adequately captured in the simulations. However, the plasma exposure after colon administration of solutions of the low-solubility drugs AZ1, AZ3, and felodipine was overpredicted.

For solutions in GastroPlus, the AAFE values for AUC_0-*t*_ and *F*_rel,colon_ were both outside the set 2-fold prediction error limit (3.63 and 2.98, respectively) and the corresponding AFE values were 0.54 and 0.53, which indicated a trend for underprediction (Table 6). The predictions of AUC_0-*t*_ and *F*_rel,colon_ were within a 2-fold deviation from the observed values in 38 and 54% of the cases, respectively, for the solutions (Table 6). For the suspensions, the AAFE values for AUC_0-*t*_ and *F*_rel,colon_ were 2.94 and 2.09, respectively, and the corresponding AFE values were 2.59 and 1.99, which indicated a trend for overprediction (Table 6). AUC_0-*t*_ and *F*_rel,colon_ were predicted within a 2-fold deviation from the observed value in 50 and 67% of the cases (Table 6). For suspensions, *C*_max_ was predicted within a 2-fold deviation from the observed values in 50% of the cases with no trend for over- or underprediction, whereas *C*_max_ for solutions was only within a 2-fold deviation from the observed values in 23% of the cases. *T*_max_ was generally overpredicted for both solutions and suspensions (Figure 3).

**Figure 4:**
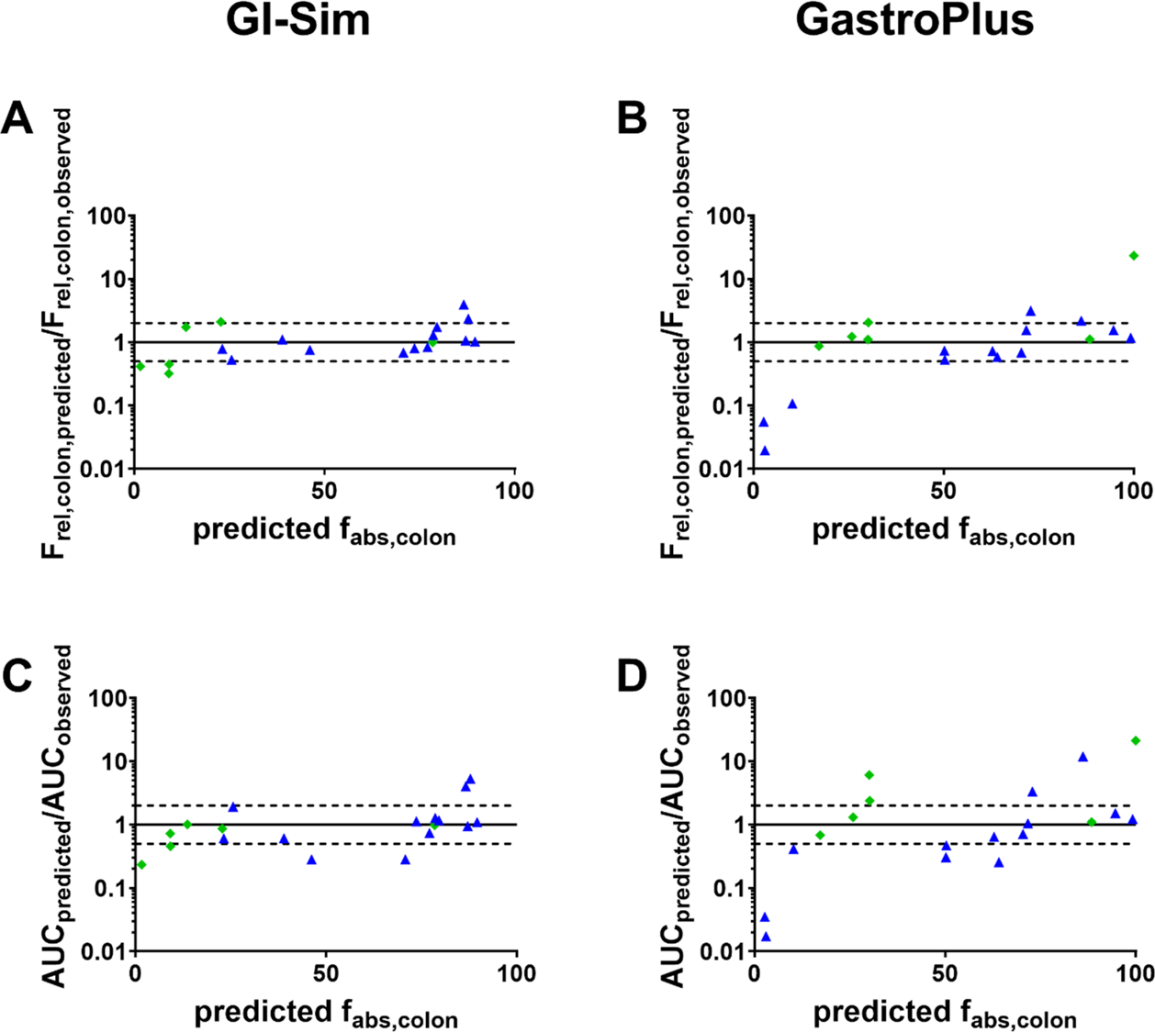
Accuracy of predicted *F*_rel,colon_ and AUC_0-*t*_ plotted in relation to the predicted *f*_abscolon_ for solutions (blue triangles) and suspensions (green diamonds). GI-Sim results are depicted in the left column and GastroPlus in the right column. The solid line is the line of unity and the dotted lines represent a 2-fold deviation.

**Figure 5:**
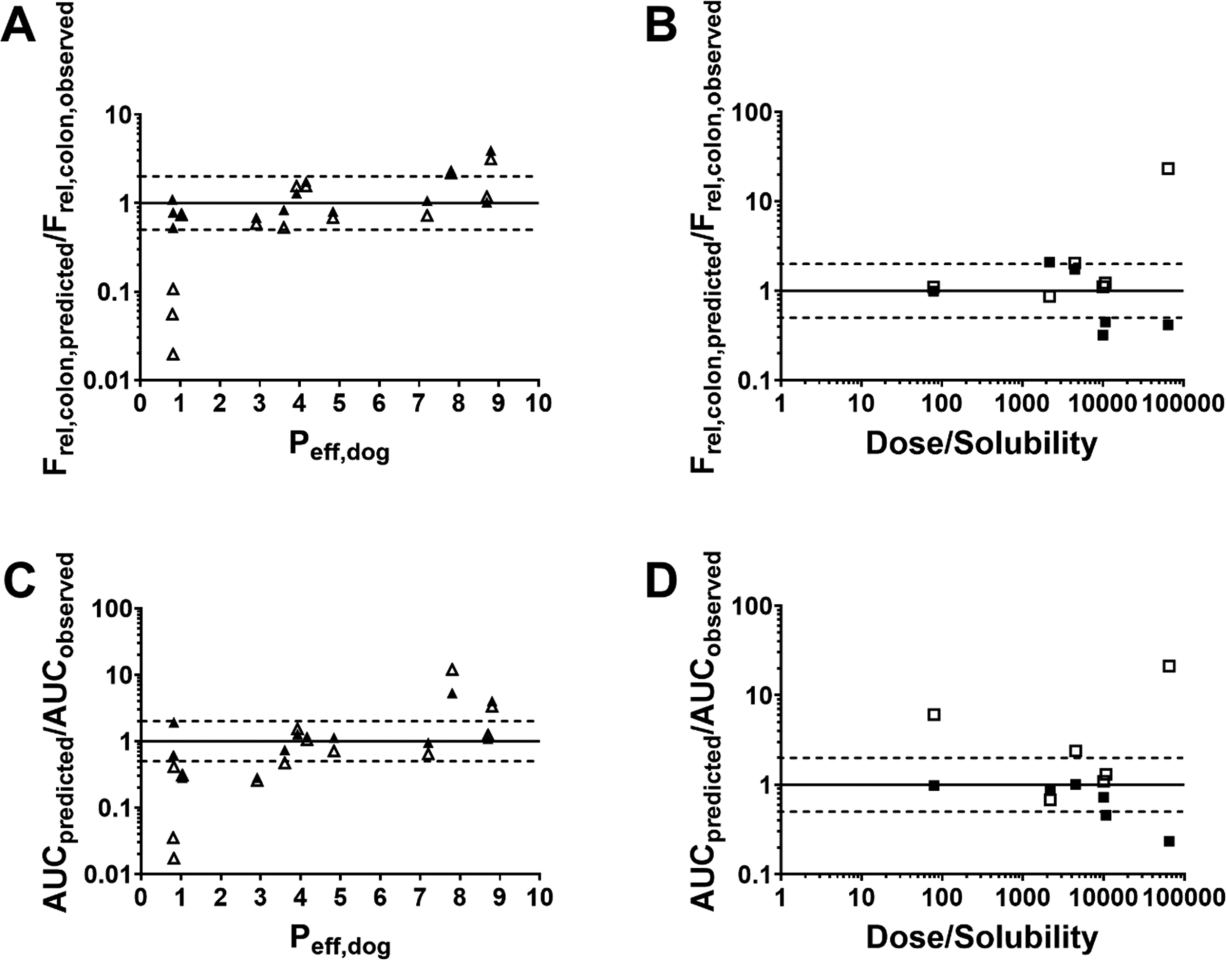
Accuracy of predicted *F*_rel,colon_ and AUC_0-*t*_ in relation to *P*_eff_ or dose/solubility ratio for solutions and suspensions. GI-Sim results are represented by black triangles (solutions) and squares (suspensions) and GastroPlus results by open triangles (solutions) and squares (suspensions). The solid line is the line of unity and the dotted lines represent a 2-fold deviation.

Any trends in prediction performance in relation to the predicted *f*_abs,colon_, *P*_eff_, or dose/solubility ratio were also investigated (Figures 4 and 5). For GI-Sim, there was no observed dependency between the prediction accuracy of the solutions or suspensions and the predicted *f*_abs,colon_, even though the predicted *f*_abs,colon_ was significantly lower for the suspensions. For GastroPlus, there was a trend for decreased prediction accuracy of the solutions at lower *P*_eff_ and predicted *f*_abs,colon_.

## 4 Discussion

The main purpose of this study was to evaluate how well the regional and colon absorption in dogs could be predicted by mechanistic PBBM using GI-Sim and GastroPlus. Regional absorption studies in dogs are performed as a surrogate for a corresponding human study for the early assessment of the extent of colon absorption, which is a critical parameter for the successful development of MR formulations. Ideally, the in vivo model would be replaced by a mechanistic in silico absorption model to reduce the use of animals and enable a more time and cost-efficient MR formulation development. However, this requires that the ability of the model to accurately predict regional/colon absorption, both qualitatively and quantitatively, is demonstrated. This was done by modeling the absorption and plasma profiles of 14 compounds with available in vivo regional and colon absorption data using an a priori approach without any fitting to observed data to reflect the real situation. Also, the evaluation was subdivided according to the formulation type, i.e., into solutions and suspensions, to investigate how permeability and solubility/dissolution rate affected the prediction performance of the models.

The extent of colon absorption of solutions was considered to be predicted with a sufficient degree of accuracy by GI-Sim since the predefined limit for accurate predictions (AAFE ≤ 2) was met and since no trend for over-/underprediction was observed. In addition, the predictive performance was not dependent on the predicted *f*_abs_ or the *P*_eff_ used (Figures 4 and 5A,C). For GastroPlus, the limit for accurate predictions was not met for either AUC_0-*t*_ or *F*_rel,colon_. The somewhat lower prediction accuracy was mainly related to an underprediction of *f*_abs,colon_ for the compounds with lower permeability, including atenolol, ranitidine, and enalaprilate. This demonstrates that the two software differ even though the overall model structure is the same. For example, in GastroPlus, the lipophilicity (Log D and log P) is taken into account when the *P*_eff_ in each compartment is calculated while GI-Sim only considers the unionized fraction.^23,26^ Changes in the colon absorption scale factors may be considered to improve the prediction accuracy for low-permeability drugs in GastroPlus, but such an evaluation was out of scope for this study. Furthermore, both GastroPlus and GI-Sim overpredicted the colon absorption for the solutions of the poorly soluble drugs AZ1, AZ3, and felodipine, which could be due to the fact that precipitation may have occurred in vivo as described earlier by Sutton.^20^ If such information would have been available and accounted for in the modeling, the observed prediction performance might have been improved for both software.

The extent of colon absorption of suspensions was considered to be predicted with a sufficient degree of accuracy by GI-Sim since the predefined limit for accurate predictions (AAFE ≤ 2) was met for both AUC_0-*t*_ and *F*_rel,colon_, but with a slight trend for underprediction. The low number of compounds administered as a suspension made it more difficult to detect any clear trends, but GI-Sim may potentially underpredict both AUC_0-*t*_ and *F*_rel,colon_ of low-solubility compounds (high dose/solubility ratios). For GastroPlus, the AAFE values for AUC_0-*t*_ and *F*_rel,colon_ were 2.94 and 2.09, respectively, and both parameters were generally overpredicted. Part of the reason for these values was the large overprediction of the extent of colon absorption of aprepitant. This compound differed from the others as it was administered as a nanosuspension, which is more complex to model. Aprepitant was better predicted by GI-Sim, which is in line with previous studies demonstrating the ability of GI-Sim to predict increases in absorption and exposure achieved with nanoformulations of poorly soluble drugs.^26^ Furthermore, the prediction accuracy of GastroPlus did not seem to be dependent on the dose/solubility ratio. Some additional considerations should be taken into account regarding the prediction accuracy for the suspensions. In some cases, *F*_rel,colon_ of the suspension was calculated using data for an oral solution as reference, which does not accurately reflect the difference of a suspension administered orally as compared to colon. Second, the compounds administered as suspensions in this study were all low-solubility compounds, making modeling of the dissolution process particularly challenging^46^

In an early risk assessment setting, the main purpose is to be able to predict potential limitations in colon absorption. Hence, even where a quantitatively accurate prediction of exposure after administration to the colon is not achieved, the ability to qualitatively predict differences in regional absorption should be considered enough to enable this risk assessment. Although there were some differences in the prediction performance between GI-Sim and GastroPlus, where the AAFE criteria were not met by GastroPlus, overall both models were able to predict regional differences in absorption as well as the AUC_0-*t*_ and *F*_rel,colon_ with acceptable accuracy in the majority of cases. It should also be taken into consideration that in this evaluation, the intention was to make the simulation conditions as similar as possible in both software. The applied methodology may not be optimal for any of the investigated software but reflects the effort to generate comparable results. With all of this in mind, the results suggest that it may indeed be possible to replace in vivo regional absorption studies in dogs in the early assessment of the risk for colon absorption limitation with the evaluated models.

One critical step in the modeling strategy was the selection of permeability value in dogs. Even though dog *P*_eff_ values have been published for some of the compounds included in this study,^5^ this is generally not the case. Both GI-Sim and GastroPlus have built-in human *P*_eff_–dog *P*_eff_ correlations, but the accuracy of the available correlations is not well-established. Therefore, in this study, three general approaches to estimate dog *P*_eff_ were evaluated and the approach, dividing the compounds into two groups depending on the human permeability class, was the most successful. The defined limit of a human *P*_eff_ of 1.34 (*P*_eff_ for metoprolol) was based on a work by Dahlgren et al., where they measured *P*_eff_ indirectly in dogs with intestinal stomas and presented data showing a higher permeability in dogs in comparison to humans for the low-permeability compound atenolol, whereas the high-permeability compounds metoprolol and ketoprofen had similar *P*_eff_ values in dogs and human.^5^ Although the exact limit is somewhat arbitrary, one could argue that, out of the approaches examined here, this is the most scientifically sound approach based on available data. Considering physiological differences in the GI tract, it is plausible that compounds with low permeability in humans may be better absorbed in dogs due to increased possibilities for paracellular transport.^47−49^ However, when passive transcellular permeability is already sufficiently high in humans, the larger paracellular pores in the dogs play a minor quantitative role. Overall, it was concluded that, since this approach offered the best predictive performance and was considered mechanistically sound, it was used to estimate dog *P*_eff_ throughout this study.

Despite the encouraging results obtained in this study, the predictive performance of GI-Sim and GastroPlus could be further improved. In addition to improving the estimation of the dog *P*_eff_ discussed above, the physiological relevance could be increased. For example, the scaling of the surface area available for absorption in the colon in GI-Sim should ideally be derived from the understanding of the dog colon physiology rather than scaled from the human model. The dog colon is known to be substantially shorter than the human colon and a direct adaption from the human model might not be appropriate.^50^ The SI part of the GI-Sim dog model could also be modified to more accurately reflect the physiology of the dog GI tract. It has been proposed that a more appropriate model should have a larger number of jejunal compartments to reflect the fact that dogs have a proportionally longer jejunum and shorter ileum than humans.^51,52^ However, this was out of scope for this study.

In this evaluation, care was taken to ensure the use of high-quality input data when available but since data was gathered from many different sources there is a significant source of variability in how the data was generated. Additionally, data was gathered from different dog breeds, but all simulations were performed using a Beagle model, which is the only dog model available in GI-Sim and GastroPlus. However, physiologies differ between different breeds and this could affect the quality of the output.^53^ Furthermore, data on mean particle size was lacking in some cases and full particle size distribution data was only available for AZ1. It is possible that more accurate predictions could have been obtained for some of the suspensions if this data had been available. Finally, it should be pointed out that the built-in human–dog *P*_eff_ conversion in GastroPlus was not tested in this study, but this is anticipated to have no or minor effects on the obtained results. An in-depth evaluation of the reasons for any difference in the prediction performance between the different models was beyond the scope of this evaluation.

## 5 Conclusions

This study shows that mechanistic PBBM approaches can be used to predict regional differences in absorption as well as the extent of colon absorption in dogs with acceptable accuracy. This indicates that it is possible to replace in vivo regional absorption studies in dogs with in silico mechanistic biopharmaceutics modeling using GI-Sim or GastroPlus in the early assessment of the risk for colon absorption limitation, which in turn facilitate early decisions to initiate MR product development or not. Furthermore, the data set used in this study is now available to use for further improvement of the in silico dog colon absorption models.

## Supporting Information Available

Evaluation of the effective permeability strategy (PDF)
