# Supplementary material for: Physiologically Based Biopharmaceutics Modeling of Regional and Colon Absorption in Dogs
Source: Mol Pharm. 2021 Mar 15;18(4):1699–710. doi: 10.1021/acs.molpharmaceut.0c01201 (PMC8041381; doi:10.1021/acs.molpharmaceut.0c01201)
Supplement: Supplementary file 1 — mp0c01201_si_001.pdf [file mp0c01201_si_001.pdf]

Supplementary Information

Three different strategies were evaluated to estimate P_{eff} in dog in this study;

1. The first approach assumed that P_{eff} is the same in dogs and humans for all compounds.
2. The second approach used the correlation incorporated in GI-Sim, which assumes that P_{eff} in dogs is approximately three times higher than the human P_{eff}. In GastroPlus, the dog P_{eff} is approximately 2.4-3-fold higher than the human P_{eff}, depending on the permeability input value. Therefore, approach 2 was considered to be representable for the default settings in GastroPlus.
3. The third strategy divided the compounds in two groups based on previous work by Dahlgren et al [5]. Their results indicate that P_{eff} is higher in dog for low permeability compounds, but that P_{eff} is similar in dogs and humans for high permeability compounds [5]. In this evaluation, a limit was set at a human P_{eff} of 1.34 (human P_{eff} of the high permeability marker metoprolol). Compounds with a P_{eff} lower or equal to 1.34 were assumed to follow the GI-Sim correlation of having a 3-fold higher permeability in dogs. Compounds with a P_{eff} above 1.34 were assumed to have the same P_{eff} in dogs and humans.

The different approaches were evaluated through simulations of 15 compounds after oral and colon administrations in GI-Sim only. A strategy was chosen mainly based on the ability to predict area under the plasma concentration-time curve up to the last measured concentration (AUC_{0-t}). The obtained results are presented in Supplementary Table 1.

Supplementary Table 1. Summary of the predictive performance of the evaluated strategies to estimate effective permeability in dogs.[a]

			Acceptable ≤2-fold	Inaccurate >2-fold	AAFE	AFE
**AUC**	Strategy 1	Per oral	67 (14)	33 (7)	1.92	0.67
		Colon	55 (12)	45 (10)	2.30	0.78
	Strategy 2	Per oral	76 (16)	24 (5)	1.74	0.91
		Colon	55 (12)	45 (10)	2.10	1.54
	Strategy 3	Per oral	76 (16)	24 (5)	1.98	0.78
		Colon	64 (14)	36 (22)	1.84	1.08

[a] Results are shown as percentage of simulations that falls within each specific accuracy level, as well as the Absolute Average Fold Error (AAFE) and Average Fold Error (AFE).
